# Transcriptomic variation of eyestalk reveals the genes and biological processes associated with molting in *Portunus trituberculatus*

**DOI:** 10.1371/journal.pone.0175315

**Published:** 2017-04-10

**Authors:** Jianjian Lv, Longtao Zhang, Ping Liu, Jian Li

**Affiliations:** 1 Key Laboratory of Sustainable Development of Marine Fisheries, Ministry of Agriculture, P.R.China, Yellow Sea Fisheries Research Institute, Chinese Academy of Fishery Sciences, Qingdao,China; 2 Laboratory for Marine Fisheries and Aquaculture, Qingdao National Laboratory for Marine Science and Technology, Jimo, Qingdao, China; Biocenter, Universität Würzburg, GERMANY

## Abstract

**Background:**

Molting is an essential biological process throughout the life history of crustaceans, which is regulated by many neuropeptide hormones expressed in the eyestalk. To better understand the molting mechanism in *Portunus trituberculatus*, we used digital gene expression (DGE) to analyze single eyestalk samples during the molting cycle by high-throughput sequencing.

**Results:**

We obtained 14,387,942, 12,631,508 and 13,060,062 clean sequence reads from inter-molt (InM), pre-molt (PrM) and post-molt (PoM) cDNA libraries, respectively. A total of 1,394 molt-related differentially expressed genes (DEGs) were identified. GO and KEGG enrichment analysis identified some important processes and pathways with key roles in molting regulation, such as chitin metabolism, peptidase inhibitor activity, and the ribosome. We first observed a pattern associated with the neuromodulator-related pathways during the molting cycle, which were up-regulated in PrM and down-regulated in PoM. Four categories of important molting-related transcripts were clustered and most of them had similar expression patterns, which suggests that there is a connection between these genes throughout the molt cycle.

**Conclusion:**

Our work is the first molt-related investigation of *P*. *trituberculatus* focusing on the eyestalk at the whole transcriptome level. Together, our results, including DEGs, identification of molting-related biological processes and pathways, and observed expression patterns of important genes, provide a novel insight into the function of the eyestalk in molting regulation.

## Introduction

Molting is an essential biological process occurring multiple times throughout the life history of crustaceans, and is essential for development, growth, and reproduction [1, 2]. Crustaceans experience rhythmic molting cycles that contain three main stages, inter-molt (InM), pre-molt (PrM), and post-molt (PoM), that are based on morphological and microstructure observations. The InM stage involves energy accumulation and muscle regeneration. During the PrM stage, the old exoskeleton is reabsorbed and a new exoskeleton is formed. In the PoM stage, water uptake leads to expansion, which is essential for growth.

A possible signaling pathway in molting regulation has been proposed, which includes triggering and summation phases, and involves the decapod crustacean molting gland [3]. Many regulatory neuropeptides and neuropeptide receptors and other crucial genes pertaining to molting have been identified, including molt-inhibiting hormone (MIH), crustacean hyperglycemic hormone (CHH), retinoid X receptor and ecdysteroid receptor (EcR) [4–8], cuticle-related enzymes (chitinases, chitin deacetylase, and chitin synthase) [9–11], and structural proteins of the cuticle [12, 13]. Although many studies focusing on crustacean molting have been carried out, our knowledge of the molecular mechanisms involved is still far from complete.

The eyestalk of crustaceans, in which the X-Organ/Sinus Gland complex (XO-SG) is located, plays important roles in crustacean molting [14]. The XO-SG complex releases a number of peptide hormones into the hemolymph, and these hormones are involved in a range of physiological activities such as molting, growth and postembryonic development in crustaceans [15]. The MIH triggers molting upstream of a signaling pathway that links MIH to the regulation of ecdysteroidogenesis in the decapod crustacean molting gland [16], which is secreted by the XO-SG complex [3, 17]. The multifunctional neuroendocrine hormones and CHH, which are also synthesized and stored in the XO-SG complex, were found to inhibit crustacean molting [18]. The crustacean eyestalk is covered by the extracellular cuticle, an important downstream target effector organ, which is thus an ideal organ to study transcriptomic changes during molting cycles.

In recent years, in order to clarify the molecular mechanisms of molting regulation, some molting-related genes in crustaceans have been identified via microarray technology [12, 13]. However, due to the limitations of the technology, hybridization probes can only be designed for limited cDNA or expressed sequence tag data, leading to a huge number of unknown genes or transcripts that cannot be detected [19]. RNA-seq via high-throughput sequencing is a powerful technology for identifying genes and has several distinct advantages, such as high throughput, higher sensitivity and the ability to detect new or unknown genes [13]. Due to the importance of the eyestalk in crustaceans, significant progress has been made in understanding the profile of transcript expression via RNA-seq over the last 2 years; however, these studies have mainly focused on development and neuropeptide mining rather than clarifying the mechanism of molting regulation [20–22].

*Portunus trituberculatus* (Crustacea: Decapoda: Brachyura), commonly known as the swimming crab, is widely distributed in the coastal waters of China, Japan, Korea and other East Asian countries [19]. The crab has become one of the most important economic species for its high nutritional value and fast growth in marine aquaculture [23]. Molt stages of the crab are divided into four basic periods depending on morphological feature observation [24]: post-molt (stage A and stage B), inter-molt (stage C), pre-molt (substage D0, substage D1, substage D2, substage D3, substage D4) and molt (stage E). Young juvenile crabs experience 8–10 moltings before sexual maturity [25], and show saltatory growth after each molting cycle. Comprehensive understanding of the molecular mechanisms of molting regulation is essential for controlling its growth and development of *P*. *trituberculatus*, which would benefit the aquaculture industry in China.

To investigate and gain a better understanding of the molting mechanism in *P*. *trituberculatus*, we created a reference transcriptome and digital gene expression (DGE) profile for single eyestalk samples in three major molting stages (InM, PrM, and PoM) using Illumina sequencing. To our knowledge, this is the first transcriptome-wide gene expression profiling of eyestalk in *P*. *trituberculatus*. This study will be a foundational resource for further studies in molting regulation mechanisms.

## Methods

### Animals

Ninety healthy swimming crabs (*P*. *trituberculatus*) between 80 and 100 days of age were obtained from Haifeng Company (Changyi, Weifang, China). The crabs used in the present study were artificial breeding animals. This species is the most common edible crustacean in China and is not an endangered or protected species. All the samples were acclimated in the laboratory (33 ppt, 18°C) for 1 week before beginning the experiment. Three molt stages were identified based on morphological features [24] and the expression level of MIH mRNA [26], which included intermolt (InM, stage C, all parts of the body were hard and the new carapace had not yet started secretion), premolt (PrM, substage D3/D4, the old and new carapaces were separated completely) and postmolt (PoM, stage A, parts of the body were flaccid). Nine male crabs from each molt stage (InM, PrM, and PoM) were selected. Then the crabs were placed in an ice bath until anesthetization (5–10 min), and eyestalk were dissected, cleaned of pigments and exoskeleton, and then stored in RNAlater (−20°C) until RNA extraction.

### RNA extraction, library preparation, and sequencing

Total RNA was extracted individually using Trizol (Invitrogen, Carlsbad, CA, USA) and then equivalent RNAs were pooled into each group. For library preparation, 3 μg RNA per group was used as input material. Sequencing libraries were generated using a NEBNext Ultra RNA Library Prep Kit for Illumina (NEB, USA) following the manufacturer’s recommendations and index codes were added to attribute sequences to each sample. Library quality was assessed on the Agilent Bioanalyzer 2100 system. The clustering of the index-coded samples was performed on a cBot Cluster Generation System using TruSeq PE Cluster Kit v3-cBot-HS (Illumina) according to the manufacturer’s instructions. Then, the library preparations were sequenced on an Illumina Hiseq 2000 platform and 100 bp single-end reads were generated.

### Assembly and annotation

The raw reads from our two previous works were used to assemble the reference transcriptome [19, 27]. The clean reads were assembled by Trinity as described previously [28], followed by TIGR Gene Indices clustering tools (TGICL) [29]. The longest assembled sequences were referred to as contigs. The reads were then mapped back to contigs with paired-end reads to detect contigs from the same transcript and the distances between these contigs. Finally, sequences were obtained that lacked Ns and could not be extended on either end [30]. Such sequences were defined as unigenes. The unigenes were annotated via public databases (Nr; Nt; Swiss-Prot; COG; and Kyoto Encyclopedia of Genes and Genomes, KEGG) by BlastX via an E-value cut-off of 1.0 x 10^−5^.

### DEG identification

The read counts were obtained by RSEM after being mapped back to the reference transcriptome, which was normalized via RPKM [30]. Prior to differential gene expression analysis, for each sequenced library, the read counts were adjusted by the edgeR program package through one scaling normalized factor [31]. Differential expression analysis of two conditions was performed using the DEGSeq R package (1.12.0) [32]. The P values were adjusted using the Benjamini & Hochberg method. Corrected p-value of 0.005 and log2 (fold change) of 1 were set as the threshold for significantly differential expression. Selected differentially expressed genes (DEGs) were clustered by the STEM Clustering Method [33].

### GO and KEGG pathway enrichment analysis

Gene Ontology (GO) enrichment analysis of DEGs was implemented by the GOseq R package, in which gene length bias was corrected. GO terms with a corrected P value of less than 0.05 were considered significantly enriched for DEGs. KEGG is a database resource for understanding high-level functions and utilities of the biological system, such as the cell, the organism, and the ecosystem, from molecular-level information, especially large-scale molecular datasets generated by genome sequencing and other high-throughput experimental technologies (http://www.genome.jp/kegg/). We used KOBAS to test the statistical enrichment of differential expression genes in KEGG pathways [34].

### Real-time PCR

To test the reliability of RNA-seq, 18 unigenes were selected to confirm the accuracy of different expression patterns. Their specific primers were designed with Primer Premier 5 software (Premier Biosoft International) (S1 Table). First strand cDNA was synthesized with prepared RNA of Illumina sequencing using a PrimeScript RT reagent kit (Takara, Dalian, China). Quantitative real-time PCR was performed using a 7500 Fast Real-Time PCR System and QuantiFast SYBR Green PCR Kit (Qiagen, USA). The PCR was carried out in a total volume of 10 μl and performed with the following thermal profile: 95°C for 5 min, 40 cycles of 95°C for 10 s and 60°C–65°C for 30 s. Fluorescence levels were measured after the 60°C step. At the end of the PCR cycles, a melt curve was generated to analyze product specificity.

## Results

### Reference transcriptome assembly and annotation

**Fig 1:**
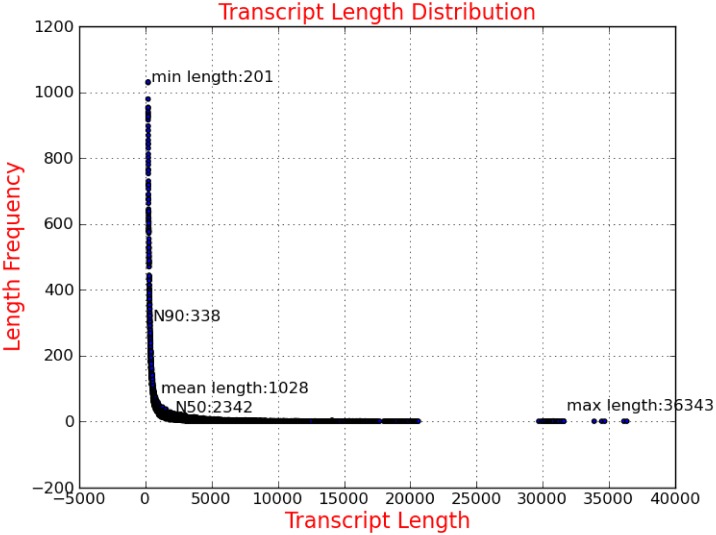
Sequence length distribution of transcripts assembled from Illumina reads.

In order to achieve a comprehensive *P*. *trituberculatus* reference transcriptome, previous RefSeq data obtained from a variety of tissues (including the eyestalk, gill, heart, hepatopancreas, and muscle) by Illumina deep sequencing was used to create the Trinity-assembled transcriptome. The overall Illumina sequencing data were deposited in the Short Read Archive database of the National Center for Biotechnology Information (NCBI) (SRR1013694, SRR1013695, SRR1013696, SRR1168416, and SRR1168417), which contained 155,713,331 read pairs and 31.02G clean bases (Table 1). Finally, we identified 186,081 transcripts with an average length of 1,028 bp (Fig 1), which contained 130,560 unigene sequences (submitted in TSA database of NCBI: SUB2309547).

**Table 1:** Summary of Illumina transcriptome sequencing, assembly and annotation for *Portunus trituberculatus*.

Raw results (after trimming)		Assembly results		Annotation results	
**Clean bases (G)**	31.02	Transcripts	186,081	Nr annotations	17,639
**Read pairs**	155,713,331	Average length of transcripts (bp)	1,028	KOG hits	16,010
**Read length (bp)**	100	Smallest transcripts (bp)	201	GO mapped	33,723
		Largest transcripts (bp)	36,343	KEGG hits	8,087

Overall, 33,723 (25.83%) unigene sequences were categorized into 58 subcategories according to GO [35]. Six subcategories, ‘cellular process’, ‘binding’, ‘cell’, ‘cell part’, ‘metabolic process’ and ‘catalytic activity’ were dominant clusters in the GO classification (Table 1; S1 Fig). A total of 16,010 unigenes could be grouped into 26 functional classes via the EuKaryotic Ortholog Groups program (KOG) [36]. Among which, ‘general function prediction’ (2,926), ‘signal transduction’ (2,488), and ‘post-translational modification, protein turnover, chaperone’ (1,720) were the dominant groups and represented 18.28%, 15.54%, and 10.74%, respectively (Table 1; S2 Fig); 8,087 unigenes were classified to 38 KEGG pathways using the KEGG database [37]. ‘infectious diseases’ (1,619 members) and ‘translation’ (1,075 members) were the two largest pathways (Table 1; S3 Fig).

### DEG identification and analysis during different molting cycles

To identify DEGs for eyestalks in different molting cycles, we sequenced three eyestalk libraries for each molting stage (InM, PrM, and PoM). Finally, the three libraries generated 14,387,942, 12,631,508 and 13,060,062 clean reads, which were mapped to the reference transcriptome and represented 74.20%, 74.10%, and 77.49%, respectively (S2 Table). The overall Illumina sequencing data were deposited in the Short Read Archive database of the NCBI (SRR5166969, SRR5166967 and SRR5166965).

**Fig 2:**
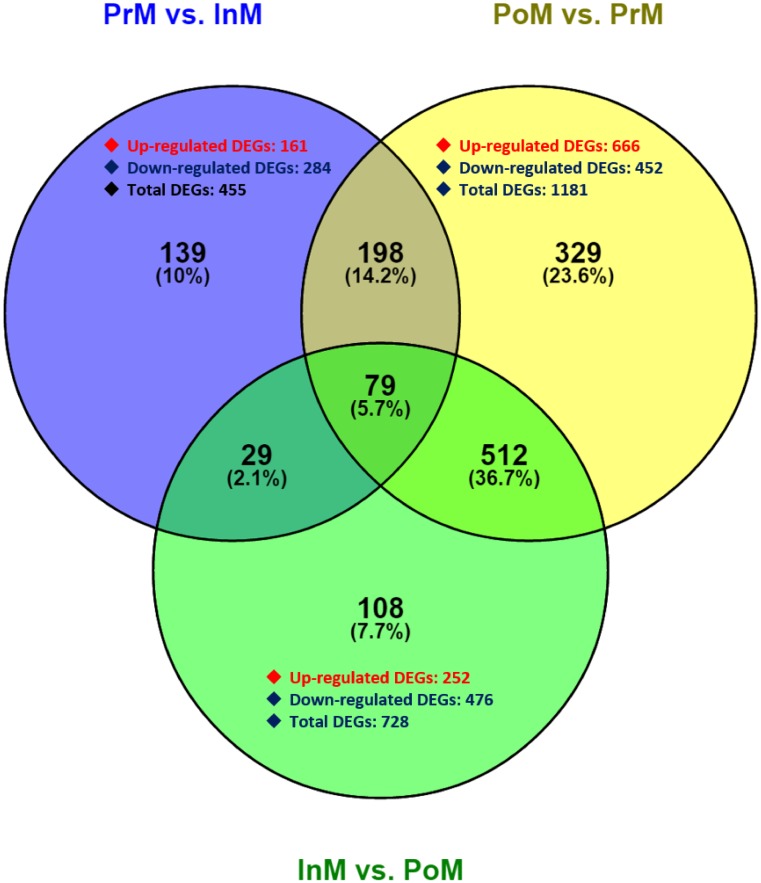
Venn diagrams of the differentially expressed genes among three molting stages.

A total of 1,394 DEGs were identified by DESeq [38]. The number of genes that were up- or down-regulated at the different molt stages is shown in Fig 2. There were 445 DEGs during the InM and PrM stages, among which, 284 were down-regulated and 161 were up-regulated in PrM stages, and 1,181 DEGs (452 down-regulated and 666 up-regulated) were identified between the PrM and PoM stages. A total of 728 DEGs (476 down-regulated and 252 up-regulated) were identified between InM and PoM.

Eighteen unigenes were subjected to qPCR assays in order to verify the results of differential gene expression analysis (Table 2). Within the 54 comparisons, 40 (74.1%) matched well with the DEG data. Although qPCR data of 14 unigenes did not match the DEG data, they showed similar trends with the Illumina sequencing in up- or down-regulation. The results validated that the majority of the DEGs identified from the DEG analysis in different molting phases were authentic.

**Table 2:** Validation of DEGs data for the selected eighteen transcripts using qPCR for relative gene expression.

Gene ID	Gene Name	Illumina sequence			qPCR		
		InM vs. PrM	PoM vs. PrM	PoM vs. InM	InM vs. PrM	PoM vs. PrM	PoM vs. InM
**comp101914_c0**	hemocyanin beta subunit 1	1.27	2.28	0.78	3.67*	2.14	0.64
**comp99362_c0**	cAMP-responsive element-binding protein-like 2-like	1.01	0.53	0.52	1.17	0.35	0.39
**comp103008_c0**	neurogenic locus Notch protein-like	0.61	0.47	0.76	0.25*	0.34	0.72
**comp98266_c0**	transferrin	0.64	0.52	0.80	0.54	0.58	0.59
**comp96475_c1**	Espin	0.82	13.37*	16.30*	0.43	9.94*	23.09*
**comp102262_c0**	calpain-c	0.70	8.73*	12.32*	0.86	9.2*	8.03*
**comp99514_c0**	transient-receptor-potential-like protein	0.48	0.01*	0.01*	0.41	0.01*	0.01*
**comp102473_c0**	Sodium/calcium exchanger 1	0.70	1.33	1.89	0.94	2.03	1.40
**comp57954_c0**	cuticle protein VER3-like	0.69	4.77*	6.84*	0.66	10.03*	5.90*
**comp85008_c0**	Chitin binding Peritrophin-A domain	0.84	6.40*	7.03*	0.50	11.09*	3.13*
**comp90850_c0**	cuticle protein CB2-like	0.31*	5981.73*	18839.83*	0.19*	8172.91*	13834.76*
**comp88516_c0**	cuticle protein CB1	0.36	6326.38*	17268.64*	0.49	1656.17*	12670.09*
**comp61465_c0**	cuticle protein	0.63	8665.93*	13646.97*	0.65	1802.63*	13425.21*
**comp36475_c0**	cuticle protein BD1	2.38	11898.15*	4996.53*	5.06*	2644.29*	4940.72*
**comp90588_c0**	HR4 nuclear receptor	0.65	3.20*	4.93*	0.37	1.48	4.02*
**comp83668_c0**	Probable nuclear hormone receptor HR38	1.59	3.88*	2.43	1.23	1.09	1.88
**comp83095_c1**	Nuclear hormone receptor FTZ-F1	1.35	6.70*	4.94*	1.65	1.94	2.99
**comp93173_c0**	Ecdysone-induced protein 74EF isoform B	0.73	0.48	0.65	0.39	0.13*	0.35

* represents a significant difference between the InM, PrM and PoM.

### GO and KEGG enrichment analysis

**Fig 3:**
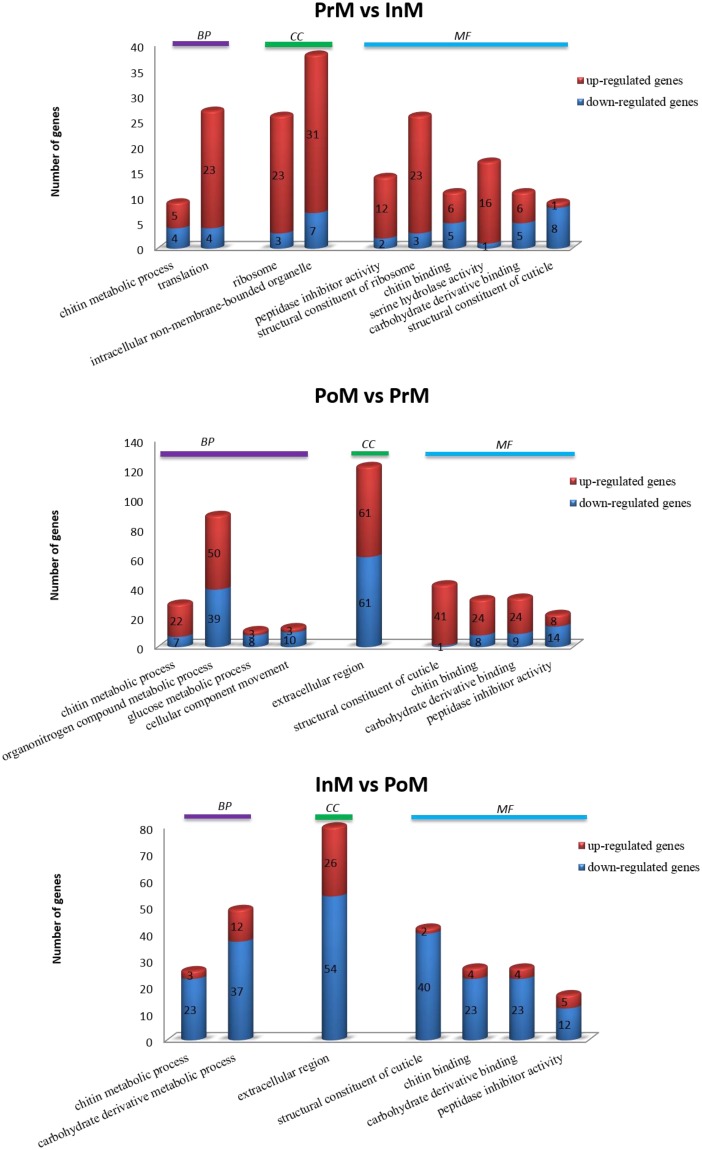
GO enrichment analysis of the differentially expressed genes.

DEGs were further analyzed according to GO functional enrichment analysis (Fig 3). The 188 DEGs of PrM vs. InM were enriched in 10 processes, among which, most of the DEGs (77.66%) were up-regulated in PrM. In the 10 processes, chitin metabolic process (GO:0006030), ribosome (GO:0005840) and peptidase inhibitor activity (GO:0030414) have the maximum enrichment level in biological process (BP), cellular component (CC), and molecular function (MF), respectively. The 393 DEGs of PoM vs. PrM were enriched in nine processes, which included 157 down-regulated and 236 up-regulated DEGs in PoM, respectively. From PrM to PoM, chitin metabolic process (GO:0006030, BP), extracellular region (GO:0005576, CC) and structural constituent of cuticle (GO:0042302, MF) were processes with the highest enrichment level. The 268 DEGs of InM vs. PoM were enriched in seven processes, among which, most of the DEGs (79.10%) were down-regulated in InM. Chitin metabolic process (GO:0006030, BP), extracellular region (GO:0005576, CC) and structural constituent of cuticle (GO:0042302, MF) were processes with the highest enrichment level between PrM and PoM.

**Fig 4:**
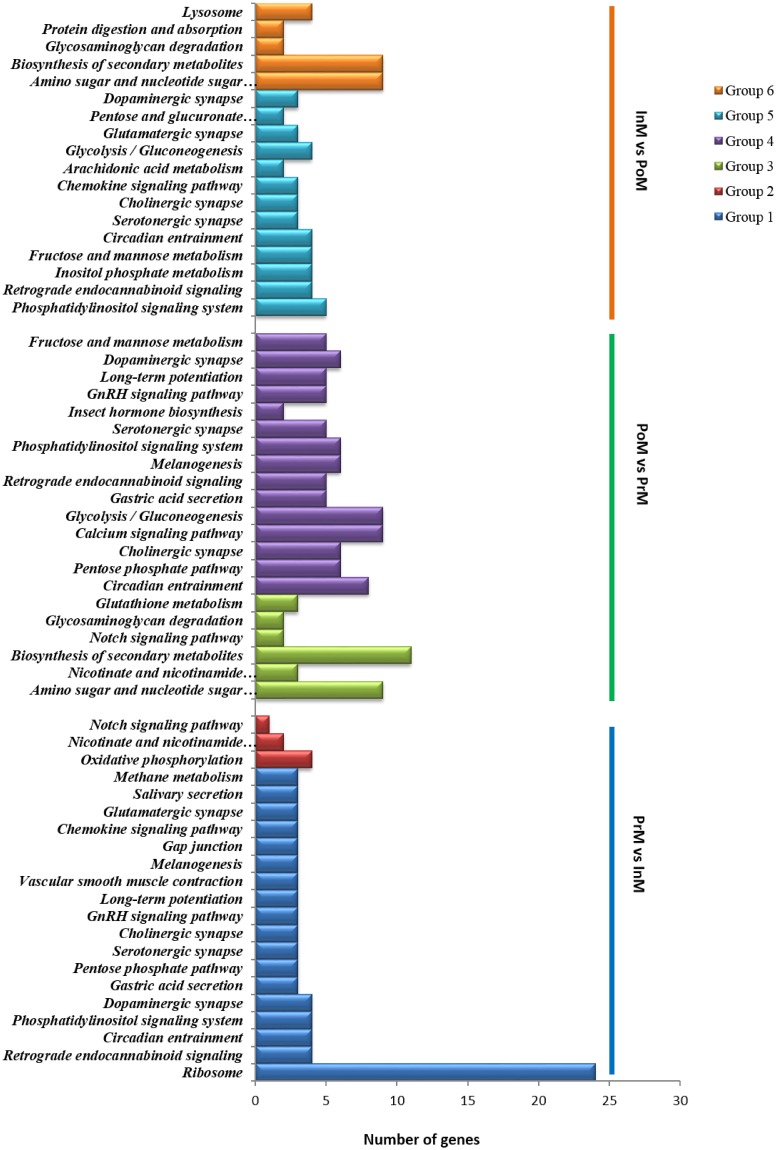
KEGG enrichment analysis of the differentially expressed genes. Group 1, DEGs up-regulated in PrM vs. InM. Group 2, DEGs down-regulated in PrM vs. InM. Group 3, DEGs up-regulated in PoM vs. PrM. Group 4, DEGs down-regulated in PoM vs. PrM. Group 5, DEGs up-regulated in InM vs. PoM. Group 6, DEGs down-regulated in InM vs. PoM.

DEGs were also analyzed according to KEGG enrichment analysis in a complete molting cycle. Six groups of DEGs were enriched in 34 KEGG pathways (Fig 4), of which DEGs up-regulated in PrM vs. InM were enriched to the largest number of KEGG pathways (18). During the PoM and PrM stages, the up- and down-regulated DEGs were enriched in six and 15 KEGG pathways, respectively. The pathway of 'amino sugar and nucleotide sugar metabolism' (ko00520) was enriched at the highest level (*p*-value = 2.43×10^−9^). Eighteen KEGG pathways were enriched during PoM and InM stages, among which, 13 KEGG pathways were up-regulated and five KEGG pathways were down-regulated.

### Expression pattern analysis of molting-related gene

**Fig 5:**
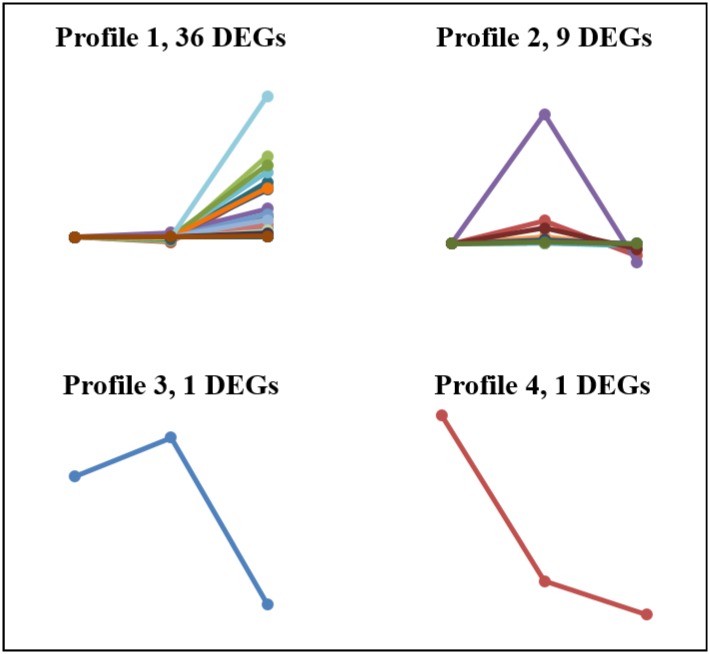
Cluster analysis of the 47 molting related genes. Forty-seven genes were grouped into four main clusters (labelled Profile 1–4) according to their expression patterns via K-means clustering. The three dots in order represented InM, PrM and PoM respectively. Lines in different colours represented different genes.

Forty-seven molting-related genes were screened and clustered by the STEM Clustering Method [33], which included 6 neuropeptide transcripts, 4 molting-related receptor transcripts, 31 cuticle transcripts and 6 chitinase transcripts. (Fig 5 and Table 3). They were grouped into four main clusters (labeled 1–4) according to their expression patterns via K-means clustering, among which, Profile 1 contained the highest number of transcripts (36, 76.6%), followed by Profile 2 (9), Profile 3 (1) and Profile 4 (1). Five neuropeptide transcripts (83.3%) were clustered in Profile 2, which were up-regulated in PrM, and then down-regulated in PoM. Most of the cuticle transcripts (29, 93.5%) and chitinase transcripts (5, 83.3%) were clustered in Profile 1, which were not significantly changed in PrM to InM and up-regulated in PoM. Four molting-related receptor transcripts showed different expression patterns, which were clustered in Profile 1 (2) and 2 (2).

**Table 3:** Information on the 47 clustered genes (DEGs).

Gene_id	Gene categories	Profile
**comp103393_c0**	cuticle	Profile 1
**comp104448_c0**	cuticle	Profile 1
**comp57738_c0**	cuticle	Profile 1
**comp94993_c0**	cuticle	Profile 1
**comp79334_c1**	cuticle	Profile 1
**comp89919_c0**	cuticle	Profile 1
**comp62755_c0**	cuticle	Profile 1
**comp99348_c0**	cuticle	Profile 1
**comp95871_c1**	chitinase	Profile 1
**comp36064_c0**	cuticle	Profile 1
**comp95871_c0**	chitinase	Profile 1
**comp89113_c1**	cuticle	Profile 1
**comp108916_c0**	chitinase	Profile 1
**comp83095_c1**	molting related receptor	Profile 1
**comp53926_c0**	cuticle	Profile 1
**comp36475_c0**	cuticle	Profile 1
**comp35636_c0**	cuticle	Profile 1
**comp101437_c0**	cuticle	Profile 1
**comp138619_c0**	cuticle	Profile 1
**comp302044_c0**	cuticle	Profile 1
**comp81349_c1**	cuticle	Profile 1
**comp81691_c0**	cuticle	Profile 1
**comp70997_c0**	cuticle	Profile 1
**comp91479_c0**	cuticle	Profile 1
**comp91757_c0**	cuticle	Profile 1
**comp86312_c0**	cuticle	Profile 1
**comp61465_c0**	cuticle	Profile 1
**comp58768_c0**	cuticle	Profile 1
**comp88516_c0**	cuticle	Profile 1
**comp78778_c0**	chitinase	Profile 1
**comp96722_c0**	cuticle	Profile 1
**comp99034_c0**	chitinase	Profile 1
**comp63340_c0**	cuticle	Profile 1
**comp90594_c0**	cuticle	Profile 1
**comp90588_c0**	molting related receptor	Profile 1
**comp104537_c0**	cuticle	Profile 1
**comp100803_c0**	neuropeptide	Profile 2
**comp103698_c3**	neuropeptide	Profile 2
**comp92243_c0**	neuropeptide	Profile 2
**comp96149_c0**	neuropeptide	Profile 2
**comp97030_c0**	neuropeptide	Profile 2
**comp86620_c0**	cuticle	Profile 2
**comp103455_c0**	chitinase	Profile 2
**comp86338_c0**	molting related receptor	Profile 2
**comp97869_c0**	molting related receptor	Profile 2
**comp90990_c0**	neuropeptide	Profile 3
**comp37103_c0**	cuticle	Profile 4

## Discussion

Molting is a vitally important metamorphosis process for *P*. *trituberculatus* and affects its physical growth, tissue regeneration, and gonad development. Apart from studies concentrating on MIH, molt hormone, and methyl farnesoate, there has been little molecular research on the molting process [4]. Our study is the first to use high-throughput filtration to investigate molting-related genes in the eyestalk of *P*. *trituberculatus* at the whole transcriptome level. Compared with other technologies, RNA-seq technology has several advantages, such as high throughput, higher sensitivity and the ability to detect new or unknown genes. This approach will assist in the discovery and annotation of novel genes that play key roles in the molting process.

The reference transcriptome used in this study was based on five groups of transcriptome data from our two previous works [19, 27], in which equal quantities of RNA were extracted and mixed from five tissues, including the eyestalk, and sequenced by Illumina. The transcript library obtained covered all of the *P*. *trituberculatus* ESTs from NCBI [27]. The overall Illumina read pairs and clean bases for all samples used in this study are 155,713,331 and 31.02G. We finally identified 186,081 transcripts after assembly analysis, the number of transcripts was significantly higher compared to the previous studies (120,137 and 141,339, respectively). Therefore, the transcriptome assembled in this study could be used as a reference transcriptome with a high coverage rate for subsequent analysis.

Identification of DEGs among the different molting phases is important to find the underlying candidate genes of molting regulation in *P*. *trituberculatus*. We detected a total of 1,394 DEGs when comparing the three important molting cycles (qvalue<0.005 & |log2 (fold change)|>1). Some genes with important roles in the regulation of molting were found in the DEGs, which included an ecdysteroid receptor (comp97869_c0) [39], chitinase (comp99034_c0 and comp108916_c0) [9, 40], hemocyanin (comp101914_c0) and nuclear hormone receptor (comp86338_c0 and comp86338_c0) [41]. Many of the DEGs have not yet been reported to have functions in the molting cycle, in spite of their having important roles in other physiological processes. For example, heat shock protein 70 (comp84939_c0), sodium/hydrogen exchanger (comp101931_c0) and methyltransferase (comp83893_c0) play an important role in immunity, osmoregulation, and epigenetic modification, respectively. This suggests that these genes might have multiple functions and may also play important roles in the regulation of molting. It also suggests that the process of molting is closely related to these physiological processes. It should be noted that most of the DEGs (746/1394) could not be aligned to any known protein by BlastX (E values less than 10^−5^). This indicates that the regulation of molting was far more complicated than we originally thought, and that there is still a long way to go in clarifying the molting mechanism in crustaceans.

GO and KEGG enrichment analysis were able to improve our understanding of the underlying biological processes related to various biological traits. A total of 15 GO processes were enriched, of which five processes (chitin metabolic process, peptidase inhibitor activity, chitin binding, carbohydrate derivative binding and structural constituent of cuticle) were found in every comparison of the three groups, which suggests these processes play an important role in molting. Thirty-four pathways were enriched via KEGG enrichment analysis. From PoM to PrM, the enriched pathways were mainly up-regulated (85.7% and 72.2% in InM vs. PoM and PrM vs. InM, respectively), which included ‘ribosome’, ‘amino sugar and nucleotide sugar metabolism’, ‘retrograde endocannabinoid signaling’, ‘circadian entrainment’ and ‘phosphatidylinositol signaling system’. The pathway of ‘ribosome’, with the highest enrichment level and the highest number of DEGs (*p*-value = 2.84461E-10, 24 DEGs), was enriched in PrM vs. InM. The ribosome is a complex molecular machine, found within all living cells, that serves as the site of biological protein synthesis (translation). Our results indicate that a large number of protein synthesis processes were triggered to prepare for molting during PrM. From PrM to PoM, 71.4% KEGG pathways were down-regulated, which suggested that the body was in a calm state after molting. However, the pathway of ‘amino sugar and nucleotide sugar metabolism’ with the highest enriched level (*p*-value = 4.14001E-08) was up-regulated in PoM vs. PrM. An amino sugar is a sugar molecule in which a hydroxyl group has been replaced with an amine group. More than 60 amino sugars are known, with one of the most abundant being N-acetyl-d-glucosamine, which is the main component of chitin. This indicated that there could be more active cuticle metabolism in the body after the early phase of PoM.

Previous studies on *Eriocheir sinensis* found a DEGs pattern associated with energy metabolism during a molting cycle. To be specific, DEGs enriched in PoM were linked to energy consumption, whereas genes enriched in InM were related to carbohydrates, lipids metabolic and biosynthetic processes [42]. However, we did not find a similar pattern with energy metabolism in our study. We believe this difference is mainly caused by the different tissues used in the two studies. Unlike the hepatopancreas, which has a main role in energy metabolism and storage, the eyestalk is a major site for the production of neurohormones and controls a variety of physiological functions such as molting and reproduction [20]. Therefore, it was not surprising that there were little energy metabolism processes or pathways enriched in DEGs in this study. But it is worth noting that a number of neuromodulator-related pathways were enriched including the dopaminergic, serotonergic, cholinergic and glutamatergic pathways, which were not found in previous research [12, 13, 42]. Hormones from the XO-SG complex appear to be controlled by the neurotransmitters/neuromodulators dopamine, serotonin, and enkephalin [43–47]. The release of some SG hormones is controlled presynaptically by dopamine, metenkephalin, and leuenkephalin, which occurs via synaptic contacts in the medulla terminalis near neuron cell bodies [48]. These neuromodulator-related pathways were up-regulated in PrM and down-regulated in PoM, which indicates molting-related neurohormone release is triggered by neuromodulators in the early phase of PrM, then aroused a series of subsequent molting processes. However, the relationship between neurotransmitters and molting-related hormones is far form clear, and further research is necessary to clarify the underlying mechanisms regulating hormone release.

The process of molting is regulated by an elaborate interplay of neuropeptide hormones, which include positive and negative regulation [13]. The signals that arise by neuropeptide hormones trigger a series of physiological activities related to molting, including losing the extracellular cuticle, escaping from the confines of the cuticle relatively rapidly, taking up water, expanding the new flexible exoskeleton and then quickly hardening it for defense and locomotion. In particular, meticulous control of regulatory proteins and genes is required to form and harden a new exoskeleton as well as to dissolve, reabsorb and shed the old one [2, 49]. Forty-seven DEGs were screened and clustered, which included six neuropeptide transcripts, four nuclear receptor (NR) transcripts, 31 cuticle transcripts and six chitinase transcripts. Most genes had similar expression patterns (Profile 1 and 2) via clustering analysis. This suggests that there is a connection between these genes throughout the molt cycle.

Crustacean neuropeptides and NRs play important roles in regulating many physiological activities, including molting, growth, feeding and reproduction [50–52]. In arthropods, the molting process is regulated by neuropeptide hormones acting via NR proteins [41]. Our results show that the highest number of these two kinds of genes were clustered in profile 2, including five neuropeptides (pigment-dispersing hormone [PDH], neuroparsins [NPs] 1 and 2, neuropeptide and neuroendocrine convertase) and 2 NRs (EcR and E75). Interestingly, the five neuropeptide transcripts were significantly up-regulated in PrM, and their functions in molting regulation have rarely been reported. For example, the PDH, which is released primarily from the eyestalk, is important in regulating pigment dispersion in crustaceans [53]. NPs were initially identified in insects with diverse roles including anti-juvenile, antidiuretic and hyperlipidemic effects [54, 55]. Neuropeptide F (NPF) is a potent stimulator regulating feeding behavior, circadian rhythm and reproduction [56–59]. The five neuropeptides were up-regulated in PrM vs. InM and subsequently down-regulated in PoM vs. PrM indicating that they play important roles in regulating molting in PrM.

EcR belongs to the NR superfamily and acts as an important transcription factor regulating downstream genes involved in the molting process [60]. Consistenting with previous studies in crustacean molting [61, 62], the expression of EcR in this research was significantly up-regulated in PrM and then down-regulated in PoM. E75, which also belongs to the NR superfamily, has been identified in numerous insects such as *D*. *melanogaster*, *Aedes aegypti* and *Galleria mellonella* [63–65]. Although the complex role of E75 is not fully understood, it was proved to be an responsive gene in ecdysteroids signaling pathway in crustacean [66, 67]. In this study, the expression pattern of E75 was similar to that of EcR, and both of them were clustered in profile 2. Our results suggest that EcR and E75 play essential roles in molting of *P*. *trituberculatus*, and point to a possible synergy between them, which deserves further verification.

Chitin, a 1, 4-β-linked polymer of *N*-acetyl-β-glucosamine, is found in crustacean shells [68]. During the pre-molt period, the epidermis secretes chitinases that degrade the inner layers of the old exoskeleton while synthesizing a new exoskeleton [69]. In this study, one of the six chitinase transcripts was clustered in Profile 1, which was up-regulated in PrM vs. InM and down-regulated in PoM vs. PrM, suggesting a specific role in degrading the old exoskeleton in the PrM stage. However, the chitinases have diverse functions in a wide range of organisms, including morphogenesis [70], host defense against chitin-bearing pathogens, cell division and sporulation [71], and regulators of development [72]. Interestingly, five of the six chitinase transcripts were clustered in Profile 1, which were significantly up-regulated in PoM, indicating they may participate in other important physiological processes after the molting stage, such as growth and development [73].

The crustacean cuticle provides initial reinforcement by cross-linking cuticular proteins attached to the cuticle chitin-fiber matrix and the protein matrices generally have very complex compositions. Thirty-one cuticle proteins were differentially expressed across the molt cycle and mainly clustered in Profile 1. The majority of them have a common variation tendency: they were not significantly changed in PrM vs. InM, but significantly up-regulated in the PoM, which shows that the majority of cuticles in *P*. *trituberculatus* were highly active at the PoM stage. The endocuticle and membranous layer form during the PoM, accompanied by calcification and sclerotization [74]. Our data suggest that the process to generate a new cuticle mainly occurs in the PoM, which is similar to other reports of crustacean cuticle protein expression during the molting cycle [69, 75].

## Conclusions

Our work is the first molt-related investigation of *P*. *trituberculatus* that is focused on the eyestalk at the whole transcriptome level. A total of 1,394 molt-related DEGs were identified. Many important processes and pathways that play a key role in molting regulation were identified including chitin metabolism, peptidase inhibitor activity, ribosome and circadian entrainment. In particular, we were the first to observe a pattern associated with neuromodulator-related pathways and four kinds of important molting-related gene families during a molting cycle. Together, our results, including screened DEGs, identified molting-related biology processes and pathways, and the observed expression pattern of important genes, provide novel insights into the functions of the eyestalk in molting regulation.

## Supporting information

S1 Fig GO classifications for the assembled unigenes. (TIF)

S2 Fig KOG classifications for the assembled unigenes. (TIF)

S3 Fig KEGG classifications for the assembled unigenes. (TIF)

S1 Table List of Differently Expressed Genes (DEGs). (XLSX)

S2 Table Summary of Digital Gene Expression (DGE). (XLSX)

## Abbreviations

CHH: crustacean hyperglycaemic hormone
DEG: different expression gene
DGE: digital gene expression
EcR: ecdysteroid receptor
InM: inter-molt
PDH: pigment-dispersing hormone
PoM: post-molt
PrM: pre-molt
NPF: Neuropeptide F
NPs: Neuroparsins
NR: nuclear receptor
USP: ultraspiracle protein
XO-SG: X-Organ / Sinus Gland complex

## References

[1] Skinner DM. 2–Molting and Regeneration Integument Pigments & Hormonal Processes. 1985:43–146.

[2] Chang ES. Physiological and biochemical changes during the molt cycle in decapod crustaceans: an overview. Journal of Experimental Marine Biology & Ecology. 1995;193(1–2):1–14.

[3] Chang ES, Mykles DL. Regulation of crustacean molting: A review and our perspectives. Gen Comp Endocrinol. 2011;172(3):323–30. 10.1016/j.ygcen.2011.04.003 21501612

[4] Techa S, Chung JS. Ecdysteroids Regulate the Levels of Molt-Inhibiting Hormone (MIH) Expression in the Blue Crab, Callinectes sapidus. PLoS ONE. 2015;10(4):19. 10.1371/journal.pone.0117278 PMC4388526 25849453

[5] Techa S, Chung JS. Ecdysone and retinoid-X receptors of the blue crab, Callinectes sapidus: Cloning and their expression patterns in eyestalks and Y-organs during the molt cycle. Gene. 2013;527(1):139–53. 10.1016/j.gene.2013.05.035 23764560

[6] Stewart MJ, Stewart P, Sroyraya M, Soonklang N, Cummins SF, Hanna PJ, et al Cloning of the crustacean hyperglycemic hormone and evidence for molt-inhibiting hormone within the central nervous system of the blue crab Portunus pelagicus. Comparative Biochemistry and Physiology a-Molecular & Integrative Physiology. 2013;164(2):276–90. 10.1016/j.cbpa.2012.10.029 23103673

[7] Hill RJ, Graham LD, Turner KA, Howell L, Tohidi-Esfahani D, Fernley R, et al Structure and Function of Ecdysone Receptors-Interactions with Ecdysteroids and Synthetic Agonists In: Dhadialla TS, editor. Advances in Insect Physiology, Vol 43: Insect Growth Disruptors. Advances in Insect Physiology 43 2012 p. 299–351.

[8] Durica DS, Chung CK, Hopkins PM. Characterization of EcR and RXR Gene Homologs and Receptor Expression During the Molt Cycle in the Crab, Uca pugilator. Am Zool. 1999;39(4):758.

[9] Rocha J, Garcia-Carreno FL, Muhlia-Almazan A, Peregrino-Uriarte AB, Yepiz-Plascencia G, Cordova-Murueta JH. Cuticular chitin synthase and chitinase mRNA of whiteleg shrimp Litopenaeus vannamei during the molting cycle. Aquaculture. 2012;330:111–5.

[10] Zhang SY, Jiang SF, Xiong YW, Fu HT, Sun SM, Qiao H, et al Six chitinases from oriental river prawn Macrobrachium nipponense: cDNA characterization, classification and mRNA expression during post-embryonic development and moulting cycle. Comparative Biochemistry and Physiology B-Biochemistry & Molecular Biology. 2014;167:30–40. 10.1016/j.cbpb.2013.09.009 24096116

[11] Tom M, Manfrin C, Chung SJ, Sagi A, Gerdol M, De Moro G, et al Expression of cytoskeletal and molt-related genes is temporally scheduled in the hypodermis of the crayfish Procambarus clarkii during premolt. J Exp Biol. 2014;217(23):4193–202. 25278476 10.1242/jeb.109009

[12] Seear PJ, Tarling GA, Burns G, Goodall-Copestake WP, Gaten E, Özkaya Ö, et al Differential gene expression during the moult cycle of Antarctic krill (Euphausia superba). BMC Genomics. 2010;11(1):582. 20958982 10.1186/1471-2164-11-582 PMC3091729

[13] Kuballa A, Holton T, Paterson B, Elizur A. Moult cycle specific differential gene expression profiling of the crab Portunus pelagicus. BMC Genomics. 2011;12(1):147. 21396120 10.1186/1471-2164-12-147 PMC3062621

[14] Hopkins PM. The eyes have it: a brief history of crustacean neuroendocrinology. Gen Comp Endocrinol. 2012;175(3):357–66. 10.1016/j.ygcen.2011.12.002 22197211

[15] De Kleijn DPV, Van Herp F. Molecular biology of neurohormone precursors in the eyestalk of Crustacea. Comparative Biochemistry and Physiology Part B: Biochemistry and Molecular Biology. 1995;112(4):573–9. 10.1016/0305-0491(95)00126-3 8590372

[16] Pitts NL, Mykles DL. Localization and expression of molt-inhibiting hormone and nitric oxide synthase in the central nervous system of the green shore crab, Carcinus maenas, and the blackback land crab, Gecarcinus lateralis. Comparative Biochemistry and Physiology Part A: Molecular & Integrative Physiology. 2017;203:328–40. 10.1016/j.cbpa.2016.10.012 27989866

[17] Webster SG, Chang E.S., Thiel M.. Endocrinology of molting Physiology. 2015;Oxford:1–35.

[18] Chung JS, Zmora N, Katayama H, Tsutsui N. Crustacean hyperglycemic hormone (CHH) neuropeptides family: Functions, titer, and binding to target tissues. Gen Comp Endocrinol. 2010;166(3):447–54. 10.1016/j.ygcen.2009.12.011 20026335

[19] Lv J, Liu P, Wang Y, Gao B, Chen P, Li J. Transcriptome Analysis of *Portunus trituberculatus* in Response to Salinity Stress Provides Insights into the Molecular Basis of Osmoregulation. PLoS ONE. 2013;8(12):e82155 10.1371/journal.pone.0082155 24312639 PMC3849447

[20] Manfrin C, Tom M, De Moro G, Gerdol M, Giulianini PG, Pallavicini A. The eyestalk transcriptome of red swamp crayfish Procambarus clarkii. Gene. 2015;557(1):28–34. 10.1016/j.gene.2014.12.001 25479010

[21] Xu Z, Zhao M, Li X, Lu Q, Li Y, Ge J, et al Transcriptome profiling of the eyestalk of precocious juvenile Chinese mitten crab reveals putative neuropeptides and differentially expressed genes. Gene. 2015;569(2):280–6. 10.1016/j.gene.2015.05.075 26095804

[22] Suwansa-ard S, Thongbuakaew T, Wang T, Zhao M, Elizur A, Hanna PJ, et al In silico Neuropeptidome of Female Macrobrachium rosenbergii Based on Transcriptome and Peptide Mining of Eyestalk, Central Nervous System and Ovary. PLoS ONE. 2015;10(5). 10.1371/journal.pone.0123848 PMC4449106 26023789

[23] Ren Q, Pan L. Digital gene expression analysis in the gills of the swimming crab (Portunus trituberculatus) exposed to elevated ambient ammonia-N. Aquaculture. 2014;434:108–14.

[24] Jie S, Dong-fa Z, Ze-hui H, Yi-zhou Q, Chun-jian W. Molt staging in the swimming crab Portunus trituberculatus. J Fish China. 2011;(10):1481–7.

[25] Junzeng X, Nanshan D, Wei L. A review of studies on Portunus trituberculatus in China. DONGHAI MARIN E SCIEN CE. 1997;15(4):60–5.

[26] WANG Chun-Jian Z D-F, QI Yi-Zhou, HU Ze-Hui, XIE Xi and SHEN Jie MOLT-INHIBITING HORMONE LEVELS AND ECDYSTEROID TITER DURING A MOLT CYCLE OF PORTUNUS TRITUBERCULATUS Acta Hydrobiol Sin. 2013;37(1):22–8.

[27] Lv J, Liu P, Gao B, Wang Y, Wang Z, Chen P, et al Transcriptome Analysis of the *Portunus trituberculatus*: De Novo Assembly, Growth-Related Gene Identification and Marker Discovery. PLoS ONE. 2014;9(4):e94055 10.1371/journal.pone.0094055 24722690 PMC3983128

[28] Grabherr MG, Haas BJ, Yassour M, Levin JZ, Thompson DA, Amit I, et al Full-length transcriptome assembly from RNA-Seq data without a reference genome. Nat Biotechnol. 2011;29(7):644–52. 10.1038/nbt.1883 21572440 PMC3571712

[29] Pertea G, Huang X, Liang F, Antonescu V, Sultana R, Karamycheva S, et al TIGR Gene Indices clustering tools (TGICL): a software system for fast clustering of large EST datasets. Bioinformatics. 2003;19(5):651–2. 12651724 10.1093/bioinformatics/btg034

[30] Li B, Dewey CN. RSEM: accurate transcript quantification from RNA-Seq data with or without a reference genome. BMC Bioinformatics. 2011;12(1):1. 21816040 10.1186/1471-2105-12-323 PMC3163565

[31] Robinson MD, McCarthy DJ, Smyth GK. edgeR: a Bioconductor package for differential expression analysis of digital gene expression data. Bioinformatics. 2010;26(1):139–40. 10.1093/bioinformatics/btp616 19910308 PMC2796818

[32] Wang L, Feng Z, Wang X, Wang X, Zhang X. DEGseq: an R package for identifying differentially expressed genes from RNA-seq data. Bioinformatics. 2010;26(1):136–8. 10.1093/bioinformatics/btp612 19855105

[33] Ernst J, Bar-Joseph Z. The Short Time-series Expression Miner (STEM).

[34] Wu J, Mao X, Cai T, Luo J, Wei L. KOBAS server: a web-based platform for automated annotation and pathway identification. Nucleic Acids Res. 2006;34(Web Server issue):W720–4. 10.1093/nar/gkl167 16845106 PMC1538915

[35] Young MD, Wakefield MJ, Smyth GK, Oshlack A. Method Gene ontology analysis for RNA-seq: accounting for selection bias. Genome Biol. 2010;11:R14 10.1186/gb-2010-11-2-r14 20132535 PMC2872874

[36] Koonin EV, Fedorova ND, Jackson JD, Jacobs AR, Krylov DM, Makarova KS, et al A comprehensive evolutionary classification of proteins encoded in complete eukaryotic genomes. Genome Biol. 2004;5(2):R7 10.1186/gb-2004-5-2-r7 14759257 PMC395751

[37] Kanehisa M, Araki M, Goto S, Hattori M, Hirakawa M, Itoh M, et al KEGG for linking genomes to life and the environment. Nucleic Acids Res. 2008;36(suppl 1):D480–D4. 18077471 10.1093/nar/gkm882 PMC2238879

[38] Anders S. Analysing RNA-Seq data with the DESeq package. Mol Biol. 2010.

[39] Shyamal S, Anilkumar G, Bhaskaran R, Doss GP, Durica DS. Significant fluctuations in ecdysteroid receptor gene (EcR) expression in relation to seasons of molt and reproduction in the grapsid crab, Metopograpsus messor (Brachyura: Decapoda). Gen Comp Endocrinol. 2015;211(17):39–51. 25448252 10.1016/j.ygcen.2014.11.006

[40] Tan SH, Degnan BM, Lehnert SA. The Penaeus monodon Chitinase 1 Gene Is Differentially Expressed in the Hepatopancreas During the Molt Cycle. Mar Biotechnol (NY). 2000;2(2):126–35. Epub 2000/05/17. 10811951 10.1007/s101269900016

[41] Nakagawa Y, Henrich VC. Arthropod nuclear receptors and their role in molting. FEBS J. 2009;276(21):6128–57. 10.1111/j.1742-4658.2009.07347.x 19796154

[42] Huang S, Wang J, Yue W, Chen J, Gaughan S, Lu W, et al Transcriptomic variation of hepatopancreas reveals the energy metabolism and biological processes associated with molting in Chinese mitten crab, Eriocheir sinensis. Scientific Reports. 2015;5. 10.1038/srep14015 PMC4570184 26369734

[43] Fingerman M, Julian WE, Spirtes MA, Kostrzewa RM. The presence of 5-hydroxytryptamine in the eyestalks and brain of the fiddler crab UCA pugilator, its quantitative modification by pharmacological agents, and possible role as a neurotransmitter in controlling the release of red pigment-dispersing hormone. Comp Gen Pharmacol. 1974;5(3–4):299–303.

[44] Webster SG, Keller R, Dircksen H. The CHH-superfamily of multifunctional peptide hormones controlling crustacean metabolism, osmoregulation, moulting, and reproduction. Gen Comp Endocrinol. 2012;175(2):217–33. 10.1016/j.ygcen.2011.11.035 22146796

[45] Robert A, Monsinjon T, Delbecque J-P, Olivier S, Poret A, Foll F Le, et al Neuroendocrine disruption in the shore crab Carcinus maenas: Effects of serotonin and fluoxetine on chh- and mih-gene expression, glycaemia and ecdysteroid levels. Aquat Toxicol. 2016;175:192–204. 10.1016/j.aquatox.2016.03.025 27060239

[46] Duangprom S, Kornthong N, Suwansa-ard S, Srikawnawan W, Chotwiwatthanakun C, Sobhon P. Distribution of crustacean hyperglycemic hormones (CHH) in the mud crab (Scylla olivacea) and their differential expression following serotonin stimulation. Aquaculture. 2017;468:481–8.

[47] Ollivaux C, Dircksen H, Toullec JY, Soyez D. Enkephalinergic control of the secretory activity of neurons producing stereoisomers of crustacean hyperglycemic hormone in the eyestalk of the crayfish Orconectes limosus. J Comp Neurol. 2002;444(1):1–9. 11835178 10.1002/cne.1426

[48] Cahansky AV, Medesani DA, Chaulet A, Rodríguez EM. In vitro effects of both dopaminergic and enkephalinergic antagonists on the ovarian growth of Cherax quadricarinatus (Decapoda, Parastacidae), at different periods of the reproductive cycle. Comparative Biochemistry & Physiology Part A Molecular & Integrative Physiology. 2011;158(1):126–31. 10.1016/j.cbpa.2010.09.016 20883810

[49] Shechter A, Tom M, Yudkovski Y, Weil S, Chang SA, Chang ES, et al Search for hepatopancreatic ecdysteroid-responsive genes during the crayfish molt cycle: From a single gene to mutagenicity. J Exp Biol. 2007;210(Pt 20):3525–37. 10.1242/jeb.006791 17921154

[50] Christie AE, Stemmler EA, Dickinson PS. Crustacean neuropeptides. Cellular & Molecular Life Sciences Cmls. 2010;67(24):4135–69. 20725764 10.1007/s00018-010-0482-8 PMC11115526

[51] Hopkins PM. The eyes have it: A brief history of crustacean neuroendocrinology. Gen Comp Endocrinol. 2012;175(3):357–66. 10.1016/j.ygcen.2011.12.002 22197211

[52] Covi JA, Chang ES, Mykles DL. Neuropeptide signaling mechanisms in crustacean and insect molting glands. Invertebr Reprod Dev. 2012;56(1):33–49.

[53] Meelkop E, Temmerman L, Schoofs L, Janssen T. Signalling through pigment dispersing hormone-like peptides in invertebrates. Prog Neurobiol. 2011;93(1):125–47. 10.1016/j.pneurobio.2010.10.004 21040756

[54] Girardie J, Boureme D, Couillaud F, Tamarelle M, Girardie A. Anti-juvenile effect of neuroparsin A, a neuroprotein isolated from locust corpora cardiaca. Insect Biochem. 1987;17(7):977–83.

[55] Badisco, Claeys L, Loy IV, Hiel TV, Franssens M, Simonet V, et al Neuroparsins, a family of conserved arthropod neuropeptides. Gen Comp Endocrinol. 2007;153(1):64–71. 17475261 10.1016/j.ygcen.2007.03.008

[56] Nässel DR, Wegener C. A comparative review of short and long neuropeptide F signaling in invertebrates: Any similarities to vertebrate neuropeptide Y signaling? Peptides. 2011;32(6):1335–55. 10.1016/j.peptides.2011.03.013 21440021

[57] Lee KS, You KH, Choo JK, Han YM, Yu K. Drosophila short neuropeptide F regulates food intake and body size. J Biol Chem. 2004;279(49):27-. 10.1074/jbc.M407842200 15385546

[58] Shang Y, Donelson NC, Vecsey CG, Guo F, Rosbash M, Griffith LC. Short Neuropeptide F Is a Sleep-Promoting Inhibitory Modulator. Neuron. 2013;80(1):171–83. 10.1016/j.neuron.2013.07.029 24094110 PMC3792499

[59] Chen W, Shi W, Li L, Zheng Z, Li T, Bai W, et al Regulation of sleep by the short neuropeptide F (sNPF) in Drosophila melanogaster. Insect Biochem Mol Biol. 2013;43(9):809–19. 10.1016/j.ibmb.2013.06.003 23796436

[60] Ecdysteroid Receptor: Springer Berlin Heidelberg; 2008 405–6 p.

[61] Hirano M, Ishibashi H, Yamauchi R, Kim JW, Arizono K. Expression Analysis of Ecdysone Receptor and Ultraspiracle through Molting Period in Mysid Crustacean, Americamysis bahia. 2008.

[62] Shyamal S, Anilkumar G, Bhaskaran R, Doss GP, Durica DS. Significant fluctuations in ecdysteroid receptor gene (EcR) expression in relation to seasons of molt and reproduction in the grapsid crab, Metopograpsus messor (Brachyura: Decapoda). Gen Comp Endocrinol. 2015;211:39–51. 10.1016/j.ygcen.2014.11.006. 25448252

[63] Segraves WA, Hogness DS. The E75 ecdysone-inducible gene responsible for the 75B early puff in Drosophila encodes two new members of the steroid receptor superfamily. Genes Dev. 1990;4(2):204–19. 2110921 10.1101/gad.4.2.204

[64] Pierceall WE, Chao L, Biran A, Miura K, Raikhel AS, Segraves WA. E75 expression in mosquito ovary and fat body suggests reiterative use of ecdysone-regulated hierarchies in development and reproduction ☆. Mol Cell Endocrinol. 1999;150(1–2):73–89. 10411302 10.1016/s0303-7207(99)00022-2

[65] Jindra M, SEHNAL e Franti, #x, Riddiford LM. Isolation, characterization and developmental expression of the ecdysteroid-induced E75 gene of the wax moth Galleria mellonella. Eur J Biochem. 1994;221(2):665–75. 8174547 10.1111/j.1432-1033.1994.tb18779.x

[66] Qian Z, He S, Liu T, Liu Y, Hou F, Liu Q, et al Identification of ecdysteroid signaling late-response genes from different tissues of the Pacific white shrimp, Litopenaeus vannamei. Comparative Biochemistry and Physiology Part A: Molecular & Integrative Physiology. 2014;172:10–30. 10.1016/j.cbpa.2014.02.011. 24556071

[67] Xie X, Zhou Y, Liu M, Tao T, Jiang Q, Zhu D. The nuclear receptor E75 from the swimming crab, Portunus trituberculatus: cDNA cloning, transcriptional analysis, and putative roles on expression of ecdysteroid-related genes. Comparative Biochemistry and Physiology Part B: Biochemistry and Molecular Biology. 2016;200:69–77. 10.1016/j.cbpb.2016.06.004. 27321874

[68] Zou E, Bonvillain R. Chitinase activity in the epidermis of the fiddler crab, Uca pugilator, as an in vivo screen for molt-interfering xenobiotics. Comparative Biochemistry & Physiology Toxicology & Pharmacology Cbp. 2004;139(4):225–30. 15683831 10.1016/j.cca.2004.11.003

[69] Rocha J, Garcia-Carreño FL, Muhlia-Almazán A, Peregrino-Uriarte AB, Yépiz-Plascencia G, Córdova-Murueta JH. Cuticular chitin synthase and chitinase mRNA of whiteleg shrimp Litopenaeus vannamei during the molting cycle. Aquaculture. 2012;s 330–333(1):111–5.

[70] Merzendorfer H, Zimoch L. Chitin metabolism in insects: structure, function and regulation of chitin synthases and chitinases. J Exp Biol. 2004;206(Pt 24):4393–412. 10.1242/jeb.00709 14610026

[71] Kuranda MJ, Robbins PW. Chitinase is required for cell separation during growth of Saccharomyces cerevisiae. J Biol Chem. 1991;266(29):19758–67. 1918080

[72] Loh JT, Stacey G. Feedback regulation of the Bradyrhizobium japonicum nodulation genes. Mol Microbiol. 2001;41(6):1357–64. 11580840 10.1046/j.1365-2958.2001.02603.x

[73] Zhu Q, Arakane Y, Banerjee D, Beeman RW, Kramer KJ, Muthukrishnan S. Domain organization and phylogenetic analysis of the chitinase-like family of proteins in three species of insects. Insect Biochem Mol Biol. 2008;38(4):452–66. 10.1016/j.ibmb.2007.06.010 18342250

[74] Andersen SO. Insect cuticular sclerotization: a review. Insect Biochem Mol Biol. 2010;40(40):166–78. 19932179 10.1016/j.ibmb.2009.10.007

[75] Andersen SO. Exoskeletal proteins from the crab, Cancer pagurus. Comparative Biochemistry & Physiology Part A Molecular & Integrative Physiology. 1999;123(2):203–11. 10.1016/s1095-6433(99)00051-3 10425740

