# Supplementary figures and images for: Transcriptomic variation of eyestalk reveals the genes and biological processes associated with molting in Portunus trituberculatus
Source: PLoS One. 2017 Apr 10;12(4):e0175315. doi: 10.1371/journal.pone.0175315 (PMC5386282; doi:10.1371/journal.pone.0175315)

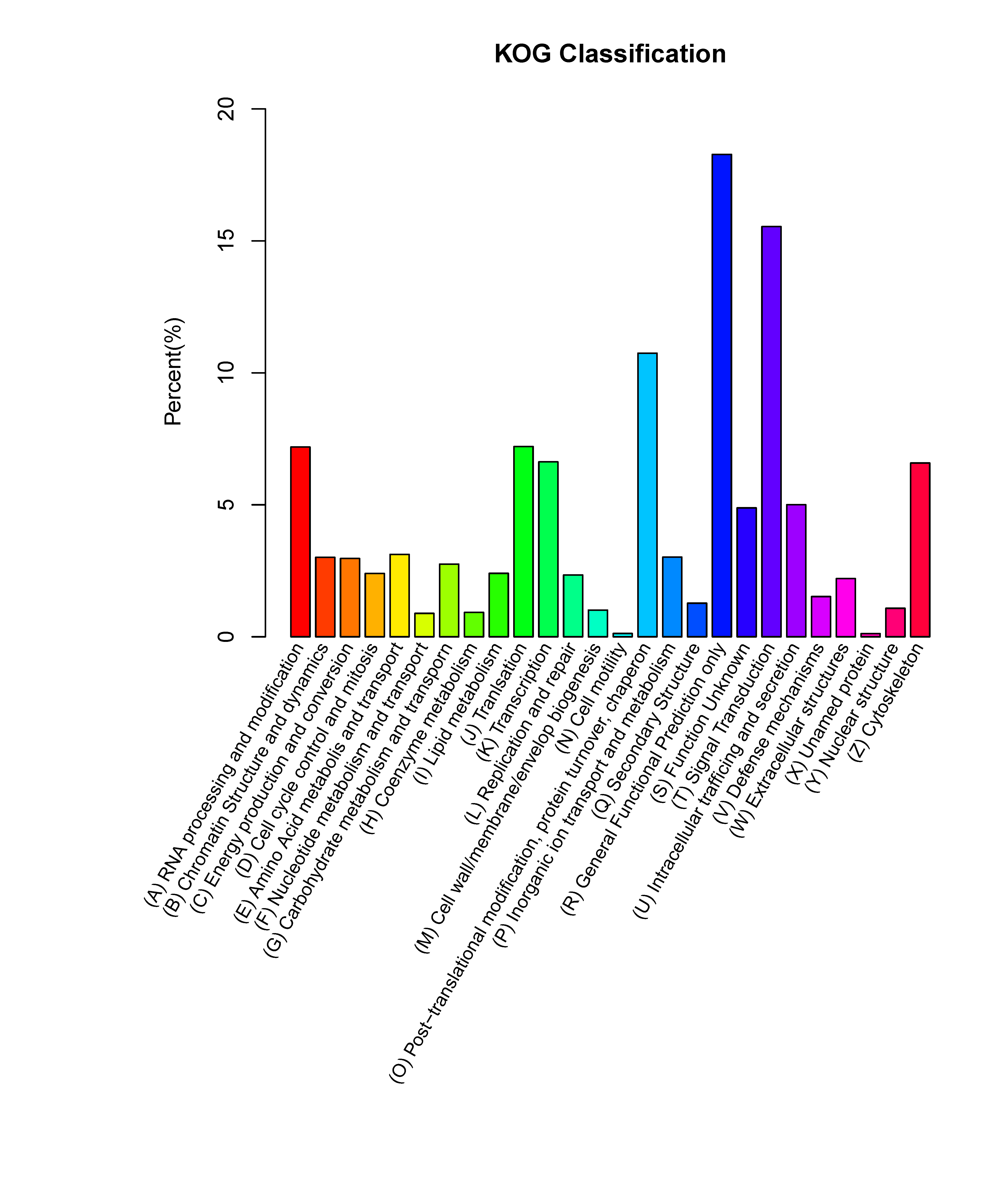

Supplement: S2 Fig — (TIF) [file pone.0175315.s002.tif]

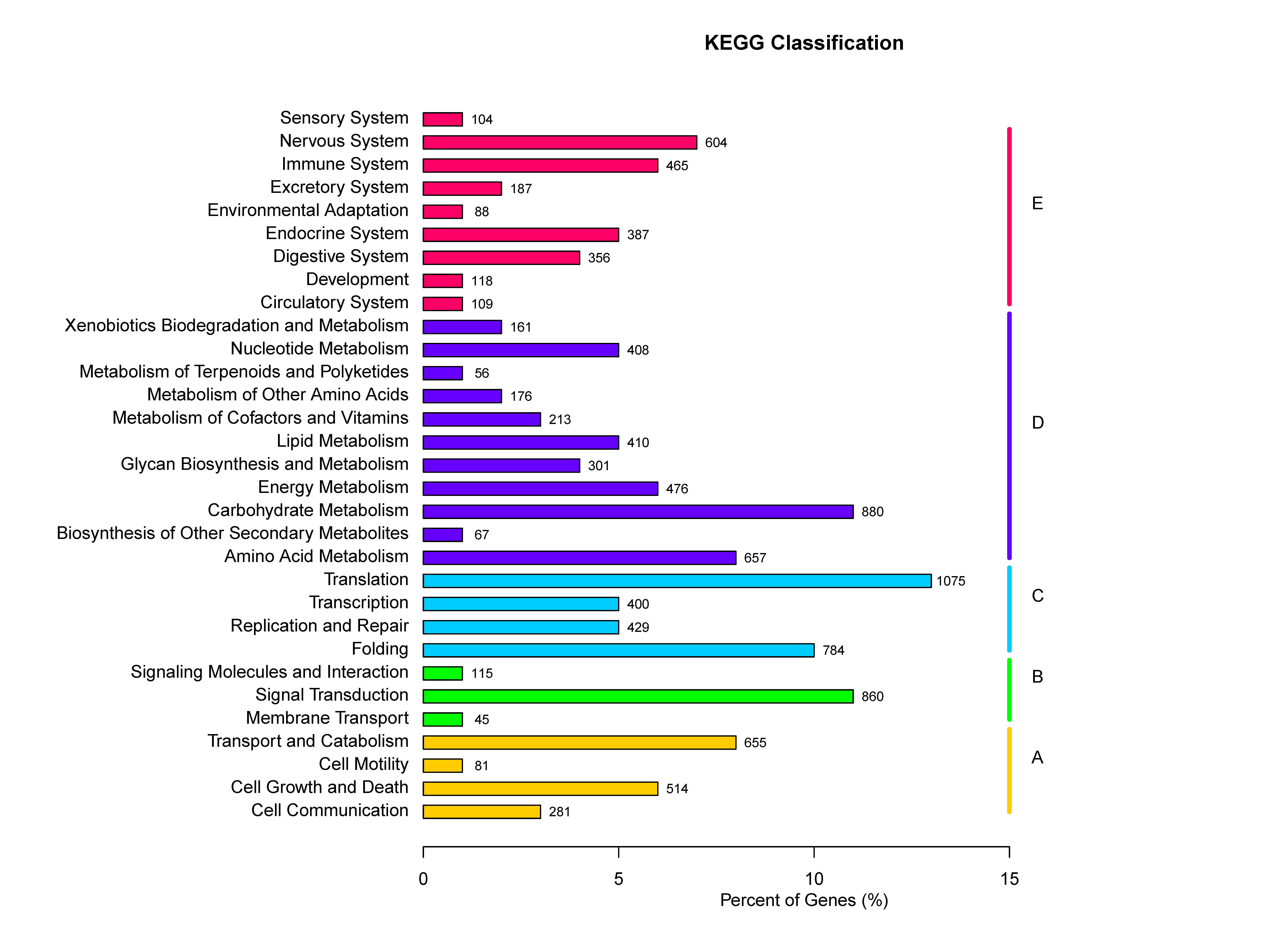

Supplement: S3 Fig — (TIF) [file pone.0175315.s003.tif]
